# Mobile Health (mHealth) Technology in Early Detection and Diagnosis of Oral Cancer-A Scoping Review of the Current Scenario and Feasibility

**DOI:** 10.1155/2022/4383303

**Published:** 2022-10-19

**Authors:** Hamad Ghaleb Dailah

**Affiliations:** Research and Scientific Studies Unit, College of Nursing, Jazan University, Jazan, Saudi Arabia

## Abstract

**Objective:**

Oral cancer is one of the most common types of cancer with dreadful consequences. But it can be detected early without much expensive equipment. Screening and early detection of oral cancer using Mobile health (mHealth) technology are reported due to the availability of the extensive network of mobile phones across populations. Therefore, we aimed to explore the existing literature regarding mHealth feasibility in the early detection of oral cancer. *Materials and Method*. An extensive search was conducted to explore the literature on the feasibility of mobile health for early oral cancer. Clinical studies reporting kappa agreement between on-site dentists and offsite health care workers/dentists in the early detection of oral cancer were included in this review. Studies describing the development of a diagnostic device, app development, and qualitative interviews among practitioners trained in using mobile health were also included in this review for a broader perspective on mHealth.

**Results:**

While most of the studies described various diagnostic accuracies using mHealth for oral cancer early detection, few studies reported the development of mobile applications, novel device designs for mHealth applications, and the feasibility of a few mHealth programs for early oral cancer detection. Community health workers equipped with a mobile phone-based app could identify “abnormal” oral lesions. Overall, many studies reported high sensitivity, specificity, and Kappa value of agreement. Effectiveness, advantages, and barriers in oral cancer screening using mHealth are also described.

**Conclusion:**

The overall results show that remote diagnosis for early detection of oral cancer using mHealth was found useful in remote settings.

## 1. Introduction

Oral cancer is one of the most common types of cancer. It refers to tumors of lips, salivary glands, tonsils, hard palate, soft palate, the floor of the mouth, and gingiva. Most of them (nearly 90%) are squamous cell carcinoma [1]. The incidence of oral cancer increases with age, and its peak is seen between 40 and 60 years [2] and shows variations in geographical distribution [3]. Globally over 400,000 new oral cancer cases are diagnosed each year, and more than half of them are reported in Asian countries [4]. The most recent estimate by the American Cancer Society for the year 2022 reported nearly 54,000 new cases of cancer in the oral cavity or oropharyngeal region and more than 10,000 deaths due to these cancers [5]. The top three cancers in these regions are breast, uterine, and lip or oral cancer, together with sharing the burden of 34% of all cancers. Over 30% of cancer mortality occurs in low and middle-income countries [4, 6]. High-risk countries in south-Asia account for more than 25% of oral cancer [4, 6]. Nearly 7.8% of the global cancer burden is shared by India [7, 8]. In Arab countries, it is prevalent in 1.8 to 2.13 per 100,000 persons, with a higher incidence in the younger population in Yemen [3]. A recent systematic review showed that in regions of Saudi Arabia, oral cancer prevalence ranged from 21.6% to 68.6% [9]. A recent report on the oral cancer burden in Arab countries estimated an incidence rate of 2.4 per 100,000 per annum and mortality in around 3,500 cases. This figure is expected to double after two decades [10]. Some developed countries, such as the United Kingdom and the Netherlands, show a considerable increase in the prevalence of oral cancer [11, 12]. A World Health Organization (WHO) report demonstrated a mortality rate of approximately 2 per 100,000 in the Middle East, lower than that present in the South Asian subcontinent and the United States [13].

Oral cancer's direct and indirect cost corresponds to the economic burden that varies widely based geographically [14]. For example, the United Kingdom has an approximate expense of $3,343 to $24,890, depending on the stage of the disease [15]. Although there are no specific studies from Saudi Arabia showing the economic burden of oral cancer, the cost of dental treatment, including oral cancer, was estimated to be around $8.33 Billion in the Middle East and North Africa (MENA) [16]. However, with one-fifth of a reduction in the advanced disease state, $30 million can be saved annually if the disease can be detected at an early stage [17]. Nearly 50% of oral cancers are diagnosed at their advanced stages (stage II and IV) with symptoms such as pain, bleeding, growth, and even lymphatic spread [18]. This is often due to patient negligence and lack of access to health care centers. When the diagnosis is delayed by one month, there is a significantly increased risk of the progress of the lesion to advanced-stage cancer [19]. Clinical and pathological staging is crucial in determining the prognosis of the case [20]. However, the present overall survival of 40% for oral cancer can be increased by more than 80% by implementing various modes of early diagnosis in the proximity of the risk community.

Fortunately, oral cancer does not require expensive equipment for early diagnosis [21]. More than 80% of them develop from preexisting precancerous lesions that can be easily noticed by the patient or a medically trained person [22]. This process is delayed because the high-risk populations have less access to health infrastructure and lack expertise in recognizing the lesions. A survey was conducted in the United Kingdom among general medical practitioners. About 97% of them revealed that they had no education in oral pathology and felt that dentists are best at screening oral cancers [23]. Besides this, in middle and low-income countries, less than 65% of the primary health care centers have access to pathological services [6, 24].

These geographical barriers are broken nowadays with the advances in the innovations in health care information technology. Some of the blooming innovations are teledentistry, mHealth, and tele-cytopathology. Teledentistry is defined as the “diagnosis and treatment of dental patients using electronically aided communication technologies” [25]. In recent times, smartphones have increased the prospects for virtual consultation, image capturing, storage, and sending of opinions to a distant location [26]. Thus the most readily available smartphones make consultation and diagnosis feasible and cost-efficient. Mobile health (mHealth) uses mobile phones and its technologies to monitor and improve health outcomes [27]. It is a health care practice that uses mobile phones and wireless devices for patient diagnosis and monitoring. Numerous applications (apps) have been developed in local languages related to health care [28]. The most widely followed method of screening oral cancers is visual and physical examination followed by palpation of the affected area and the associated lymph nodes [29]. Oral cytology is the most commonly employed screening mode for a large high-risk population [30]. Now easy-to-use imaging devices with autofluorescence imaging can detect invisible lesions and outpower the sensitivity and specificity of the conventional examination that requires a visible lesion in the oral cavity [31]. In regions wherein skilled human resources are deficient, automated analysis of tele-cytopathology can be imparted as a point of care [32].

**Figure 1:**
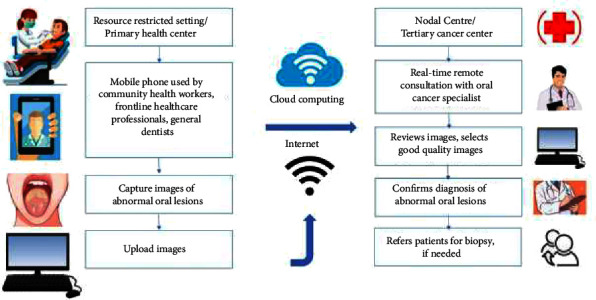
Schematic diagram showing mHealth technology in early diagnosis of oral cancer.

The concept of teledentistry is being investigated in various studies to improve disease surveillance and provide remote specialist consultation. Now, the availability of the extensive network of mobile phones across populations of all socioeconomic strata makes it a logical mode to develop various systems of mHealth for screening and early detection of oral cancer.  Figure 1 shows a schematic diagram of mHealth in the early detection of oral cancer. Some clinical studies report the applicability of mHealth for the early detection of cancer [28, 29, 33]. It is also being recommended by specialists for early detection in remote settings [34]. Therefore, we aimed to explore the existing literature regarding mHealth feasibility in the early detection of oral cancer. Our objectives are to identify clinical studies reporting the use of mHealth for oral cancer in early detection, discuss the effectiveness of mHealth approaches in cancer screening, and identify barriers and limitations in oral cancer screening using mHealth.

## 2. Methodology

This literature review was conducted to explore the literature on the feasibility of mobile health for early oral cancer. Articles up to April 25, 2022, were searched to select appropriate studies using the following PCC framework: *Population*: patients with suspicious/premalignant/malignant oral lesions including cancer; *Concept*: application of mobile health; *Context*: early detection of oral cancer. Clinical studies reporting kappa agreement between on-site dentists and offsite health care workers/dentists in the early detection of oral cancer were included in this review. Studies describing the development of a diagnostic device, app development, and qualitative interviews among practitioners trained in using mobile health were also included in this review for a broader perspective on mHealth. However, commentaries, review articles, studies describing cancers other than oral cancer, editorials, letters to the editor, conference papers, consensus papers, and questionnaire studies were excluded.

### 2.1. Search Strategy and Eligibility Criteria

Popular literature databases such as MEDLINE/PubMed, Web of Science, Scopus, and Google Scholar were extensively searched on April 25, 2022. The search was conducted based on the main three concepts (mHealth, oral cancer, and early detection) of the research question. Articles that contained the MeSH terms, keywords, and other free terms related to “mHealth,” “oral premalignant lesion,” “oral cancer,” and “early detection” were included in the preliminary screening. In addition, references to relevant studies and manual searching also were done for other potentially appropriate publications.

Forty-six articles were found in the preliminary search, out of which 25 were excluded during the title and abstract screening based on eligibility criteria. Duplicate articles were excluded with the help of a citation/reference manager (Endnote version 9). Two reviewers examined the remaining 21 articles in full length. This was based on the inclusion and exclusion criteria. In case of disagreement, a third reviewer was contacted who resolved the differences through discussion, and a final consensus was reached to include all these studies.  Table 1 shows those clinical studies reporting mHealth in oral cancer screening.

The relevant data was selected and entered into an excel sheet by two reviewers similar to the study selection. A third reviewer was involved if needed. The author's name, year of publication, country, the study's objective, number of participants, results, and conclusion were charted for all the included studies. The data obtained through data extraction were used for the qualitative synthesis of results to identify various clinical studies reporting the use of mHealth for early oral cancer detection. These are presented in the next section. The effectiveness of mHealth approaches and their barriers and limitations in oral cancer screening has been described in the discussion section.

## 3. Results

**Figure 2:**
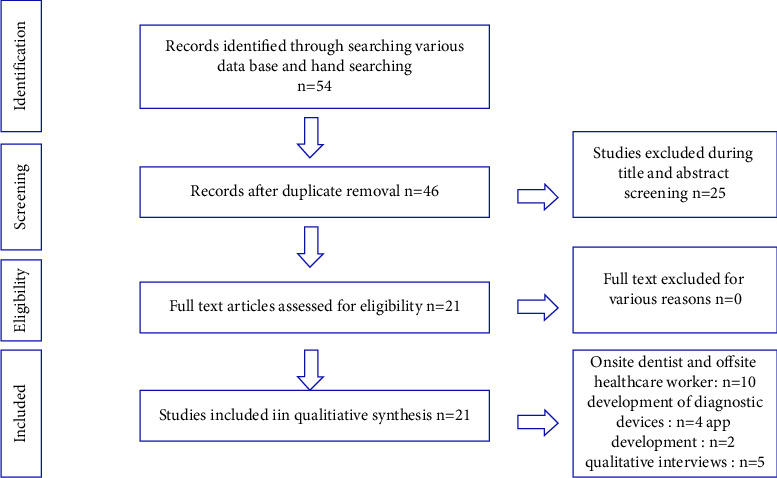
PRISMA flowchart for selection of studies.

Forty-six articles were identified during the preliminary search, out of which 21 studies matched the selection criteria. Among these, 10 studies describing the agreement between on-site dentists and offsite health care workers/dentists in the early detection of oral cancer were included in this review. Studies describing the development of diagnostic devices (4), mobile application (app) development (2), and qualitative interviews (5) among practitioners trained in using mobile health were also included in this review (Figure 2).

### 3.1. Characteristics of Clinical Studies Using mHealth for Oral Cancer Early Detection

Different study designs were used in the studies included in this scoping review. Community health workers equipped with mobile phone-based apps were used to determine the diagnostic accuracy of mHealth [34, 35, 38]. The overall results show that remote diagnosis for early detection of oral cancer using mHealth was found useful in remote settings [34, 42].

Clinical diagnoses of oral lesions made by community health workers using a mobile phone app and trained dentists by clinical examination were compared in a recent study [35]. The sensitivity and specificity of the clinical oral examination done on 100 participants showed a very high agreement (*κ* value = 0.9 (*P* < 0.001)). High sensitivity of 96.69% (95% CI, 94.15%-98.33%) and the specificity of 98.69% (95% CI, 97.52%-99.40%) was also observed. A perfect agreement between community health workers and dentists was found for erythroplakia and malignant neoplasm (*κ* = 1.0). A somewhat similar agreement was seen in the case of leukoplakia, oral lichen planus, ulcers, oral submucous fibrosis, and tobacco pouch keratosis (*κ* = 0.9).

Another mobile application called *MeMoSA* (Mobile Mouth Screening Anywhere) was reported in a prospective study involving 355 patients. Clinical diagnosis of oral lesions and decisions for a referral made by specialists were compared with those made using remote diagnosis with the help of *MeMoSA* [36]. A moderate agreement was found between clinical examination and *MeMoSA* in terms of detecting the presence of an oral lesion (*κ* = 0.604), while a higher agreement (*κ* = 0.892) was found in determining if the oral lesion was potentially malignant and if there was a need for referral to specialist. This system using *MeMoSA* showed high sensitivity of 92% and a moderate specificity of 67.5% in identifying oral lesions. However, higher sensitivity and specificity of 94% and 95.5% were seen in determining the need for further referral [36].

Artificial intelligence has also been used to identify potentially malignant oral lesions [44]. The deep learning-based classification model *MobileNet* for processing the images and real-time classification has been reported in resource-restricted areas irrespective of internet connectivity [44]. This study used a dual-mode oral cancer screening system based on a smartphone to run the deep learning-based technique. This uses intraoral autofluorescence imaging and white light imaging which was earlier found to improve the diagnostic accuracy of oral cancer image classification. These images were classified based on the deep learning model deployed on the cloud server. Specialists available at a remote place could log in to this program and provide diagnoses through a web browser. The use of *Mobilenet* to convert the standard convolutional layers into a more manageable format was found to reduce the computational cost and the model size. This proposed method showed high (81%) accuracy, sensitivity (79%), and specificity (82%) when tested on a standalone dataset.

Screening of suspected oral cancer patients by two community health workers followed by clinical evaluation by an oral diagnostician has been reported [38]. A questionnaire related to risk assessment was distributed among participants through their mobile phones. The clinical diagnosis made by the oral diagnostician was ascertained by histopathology findings. Images taken during the clinical examination were uploaded to data in an *Open Medical Record System* to be reviewed by a remote oral diagnostician. A substantial level of agreement (*k* = 0.62) was found between the community health workers and remote oral diagnosticians in detecting oral lesions (area under curve = 0.562), and a significant association between these examiners (*χ*^2^-value = 25.457; *P*-value <0.001). There were 15.31% false-positive and 2.7% false-negative cases when the findings of remote oral diagnosticians and on-site oral diagnosticians were compared. Near perfect agreement was seen between these two examiners groups (*κ* = 0.94) and a significant association between these examiners (*χ*^2^-value = 119.01; *P*-value <0.001).

Application of Artificial Neural Network (ANN) in combination with a tele-cytology was reported by Sunny et al. [39] for early detection of oral potentially malignant and/or malignant lesions. In this study, the ANN-based risk-stratification model was applied and compared with conventional cytology (*n* = 82) with the help of a portable, automated tablet-based tele-cytology platform (*Cellscope*). An overall high accuracy (84% to 86%) and agreement were reported (*κ* = 0.67 to 0.72). However, a low sensitivity (18%) was found in identifying oral premalignant lesions when tele-cytology was compared to histopathology. However, the authors expected an improvement of 15% in sensitivity while sample size calculation, an overall 30% increase was found in the accuracy when the ANN-based risk stratification model was integrated. This platform also showed high sensitivity to detecting malignant lesions (93%) and high-grade oral premalignant lesions (73%).

Photo messaging service using *WhatsApp* was reported in a primary care setting (*n* = 131) in India for remote screening of potentially malignant oral lesions [40]. There was a moderate agreement/reliability between two examiners (*κ* = 0.68 and 0.67) in identifying normal and abnormal oral lesions based on photo messaging and clinical oral examination. A high sensitivity (98.5% and 99.04%) and specificity (72% and 64%) were found for each examiner. Slightly less reliability (*κ* = 0.59 and 0.55) for the two examiners was found in an exact clinical diagnosis match based on photo messaging and clinical examination. In this scenario, a similar sensitivity (98.1% and 98.7%) and a slightly lower specificity (64% and 52%) were found for the two examiners, respectively.

Yet another study reported the concordance between clinical diagnosis and remote diagnosis and referral decisions in terms of might risk of oral lesions [42]. A moderate to strong agreement (*κ* = 0.64 to 1.00) was found to determine the presence of a lesion, the category of the lesion (oral premalignant lesion or not), and making referral decisions. A good overall sensitivity (70%) and a perfect specificity (100%) were found in this study. The false-negative rate decreased as the camera resolution increased. There was a complete agreement among all dentists regarding the applicability of teledentistry for the early detection of oral mucosal lesions [42].

*Oncogrid*, a mobile phone-based remote oral cancer surveillance program, aided early oral cancer detection [33]. This compared the concordance in identifying suspicious oral lesions, capturing interpretable images, and the need for biopsy between primary care dental practitioners, frontline health care workers (FHW), and oral cancer specialists. In the targeted cohort (*n* = 2000 screened by FHW), a positive predictive value of 45% was found. About 38% were judged to be noninterpretable. In the opportunist group (*n* = 1440), there was a 100% positive predictive value and diagnosis match of dental professionals' interpretation.

*Poi mapper* mobile application empowered health workers with a decision-based algorithm based on the risk stratification of tobacco habits [43]. A group of factory employees (*n* = 1357) with pure tobacco chewing habits were screened in two phases. In the initial phase, oral lesions were photographed after being identified. In the later phase, the remote diagnosis was established by an oral medicine specialist based on the clinical photographs. Seventy-one subjects required biopsy and the histopathology results of most cases showed hyperkeratosis and mild dysplasia, with one case of moderate dysplasia [43].

### 3.2. Studies Reporting the Development of Mobile Applications for Early Oral Cancer Detection

Two studies reported mobile application development for oral cancer detection and rehabilitation. *Prayaas* was developed for base-model mobile phones that could operate with and without the internet [28]. Its design facilitated its use for patients and health providers, which was piloted by 50 workers at a factory in rural India. Awareness regarding oral cancer and various treatment options was provided as pictures and videos for this app which was very well [28]. The majority of the participants found the app to be user-friendly (88%) and increased their knowledge about the significance of self-examination (98%). Another study by Gomes et al. developed an application for Android phones using JAVA [41]. Fifty-five high-risk patients for oral cancer were evaluated using this application to estimate various diagnostic accuracies of clinical oral examination given by two blinded dental specialists. The sensitivity of diagnosis performed by the two trainers examiners based on video recordings of the oral examination was found to be 82% to 100% (average 91%). The specificity of this diagnosis was found to be 81% to 100% (average 90.5%) with a moderate Kappa agreement value (0.597) when gold-standard and the examiners were compared.

### 3.3. Novel Device Designs for mHealth Application for Early Oral Cancer Detection

A smartphone-based oral cancer screening device enabled with neural network classification that could be used as a point-of-care device in low-resource communities has been developed by Uthoff et al. [31]. This dual-modality and dual-view device synchronize external light-emitting diode (LED) illumination that functions on a smartphone platform. It uses autofluorescence imaging (AFI) and white light imaging (WLI) to capture images of premalignant and malignant oral lesions and upload them to a cloud server for diagnosis by a remote specialist. This is done using a web app and can transmit recommendations back to the device and patient. This algorithm could clearly discriminate nearly half of the image pairs into “suspicious” and “not suspicious” with high diagnostic accuracies ranging from 81.25% to 94.94%. The ROC curve for the CNN had a high value (area under curve = 0.908). Another study by the same research groups reported a smartphone-based intraoral probe with a small and flexible imaging head that enables AFI and WLI (polarized) in a compact area with the help of a USB-connected camera module [45]. This minute flexible head enables better imaging in areas of greatest risk for cancer, such as tonsils and the base of tongue. The diagnosis made with a cloud-based remote oral diagnostician and CNN makes it feasible to be used for a remote community screening of oral cancer and home use.

An automated tablet-based microscope built on the mobile phone has been developed to screen oral premalignant and malignant lesions [46]. Here, conventional brush biopsy combined with a modified staining protocol and a tablet-based mobile microscope enables remote diagnosis by clinical specialists. This new technique shows concordance with conventional methods and enables remote diagnoses. Diffuse reflectance spectroscopy (DRS) can noninvasively quantify the optical properties of epithelial tissues to detect abnormal tissues. Hence, a portable DRS device was designed to detect oral lesions [47]. This had an innovative smart fiber-optic probe to decrease the size and power expenditure. This can be incorporated into a smartphone-based spectrometer [47].  Table 2 shows these studies mentioning the development of device developments.

### 3.4. Feasibility of Other mHealth Programs

A rural program called the “mobile-Health model,” where frontline health workers were empowered for early detection and connected to a specialist through mHealth, was found to be very effective in reducing the cost of oral cancer screening to below $1 per person [48]. The authors trained frontline health professionals to examine the oral cavity capture images using mobile phones and conduct risk factor analysis among cohorts (*n* = 42754) belonging to various resource-constrained areas between 2010 and 2018 [48]. The authors of another study ran training sessions with a mHealth prototype. A total of 8,686 people were screened through the mHealth intervention with a positivity rate of 5% for oral cancer [49]. The mHealth prototype was very acceptable to community health workers, as they thought it made screening more reliable and had a positive effect on their social standing [49].

The feasibility of using *MeMoSA* for early detection of oral cancer and communication between general dental practitioners and specialists for treatment decisions were evaluated [50]. It was found that MeMoSA was effective in the early detection of OC, identification of abnormal oral mucosal lesions, and enhanced communication with specialists [50].

Extension for Community Health Outcome (ECHO) is a cost-effective training model for regions with resource constraints [51]. A study reports the training of health care professionals using the ECHO platform and cancer screening. This enabled in to develop the knowledge and skillset necessary to conduct cancer screening in their own communities. A remarkable improvement in knowledge level scores from an average of 6.3 to 13.7 on a 15-point scale was seen immediately after the 3-day training program, and this score further increased to 14.4 after 6 months [51]. On the other hand, oral cancer screening knowledge showed no change among physicians with the newly developed mobile application (M-OncoED) [52].  Table 3 shows qualitative studies reporting the suitability of mHealth for early screening of oral cancer.

## 4. Discussion

This review identified clinical studies that reported the use of mHealth for early oral cancer detection. The selected studies showed an overall good agreement between the clinical oral examination and remote diagnosis of suspicious oral lesions using various mHealth technologies despite heterogeneity in the methodologies of these studies. It was also found that mobile phones were useful in the early detection of premalignant or malignant oral lesions in the community setting.

### 4.1. Effectiveness of mHealth Approaches in Cancer Screening

Mobile-based diagnosis of oral cancer reduces the shortcomings of conventional oral cancer screening techniques [38]. mHealth technique also empowers the local community health workers and provides timely patient referrals [38]. In most studies, community health workers identified oral lesions through a visual examination and followed up with participants who added potentially malignant oral lesions [35]. However, some found it to be time-consuming and highly dependent on the findings provided by community health workers [38]. In case the images captured were from an inappropriate site or were of poor quality, the actual lesion could be missed [38]. The diagnostic accuracy of remote consultation can be improved with the proper training of these examiners [38]. Studies also found that smartphones can be useful for patient education and remote monitoring of patients [28, 53].

Overall, a reasonably good agreement was found between both the examiners between the diagnosis made on visual and oral examination and based on images. The oral lesions were classified as normal and abnormal [40]. However, when the examiners had to give an exact diagnosis, the level of agreement between the examiners decreased to moderate [40]. These results are in line with earlier studies that based screening of oral lesions on digital images [42, 54]. This could probably be due to the difference in the nature of the oral lesions, the need for palpation during clinical examination, and its complete examination to ascertain the diagnosis was not possible through the examination of digital images [54]. The diagnostic accuracy based on images could be underestimated. The success of mHealth-based studies also depends on many factors, such as the quality of photograph efficiency of the examiner in taking high-quality photos and the cost-effectiveness of mHealth [33]. Cameras with higher resolution gave lower false-negative rates and higher concordance rates [42]. Most of the studies showed sensitivity scores similar to WHO's standard of reference of 0.85 to 0.90 [55].

Heterogeneity among studies that have studied the use of mHealth in the early detection of oral cancer occurred due to many factors. For example, heterogeneous oral white lesions with potential for malignancy were included in different studies. Another factor was the two-dimensional nature of the digital image. This could affect the diagnostic decisions made based on these images. The boundaries and texture of the lesions could have been underestimated if insufficient lighting was present while evaluating these oral premalignant lesions. Heterogeneity could also be due to the differences in the efficiency of the examiner in taking high-quality photos or the different specifications of cameras used in these studies. In studies where the phone was covered due to concerns about infection control, the image quality could have been compromised.

Poor Internet connectivity emerged as a major issue with mHealth. Keeping this in mind, connectivity and image sizes of altered mucosa of the oral cavity can be optimized to 214 to 245 KB with a resolution of 1880^*∗*^1056 [38]. Some studies describe techniques that use mobile phones with or without the internet on both Android and iOS-based devices [28]. Coughlin et al. report the acceptability of mobile apps among patients and the general public [56]. Advanced technologies such as AI were used [37] to improve the detection of oral cancer lesions outside at a low-cost even when internet connectivity was poor. The study by Song et al. [37] offers a solution to the usual complex deep learning models in the form of mobile platforms that are small and easy to operate. They also have high-performance computing capabilities of hosting an artificial intelligence cloud serve, uploading the image to the cloud server for processing, and then downloading the result with the help of *Mobilenet*, similar to the results [31]. A seamless internet connection is a bottleneck that has been solved by *Mobilenet* [37].

### 4.2. Advantages of the mHealth System

The advantages of the mHealth technique are many. The digital images collected by the dentist could be stored and used for further evaluation during follow-up [35]. The use of mobile phones with internet connections increased the availability of data for researchers [35]. mHealth app helped digitalize the clinical records that offered an opportunity for the specialist tumor to monitor the changes in the lesion over time [36]. The digital method also helps in cataloging all the captured data, which is beneficial for the clinical management of the patient [36]. Teleconsultations/remote consulting can also increase access to specialists in resource-restricted regions with a high number of cases of premalignant lesions and overall cancer [36]. In countries like India and Sri Lanka, the height number of community health workers available in the rural area [49, 57] can use their health to improve referral accuracy. This can be a cost-effective way to reduce the delay in diagnosing and offering treatment to oral cancer patients [58]. This technique can eliminate travel time and cost and unwanted referrals of patients [38].

Mobile phones were useful in individual cases and the early detection of malignant oral lesions in the community setting [42]. The user-friendliness of mobile phones also helped connect dentists with oral specialists whenever they were in doubt. Time taken was about 10 minutes to take five measures, and all the patients who operated with the dentist [42]. Another five minutes per case was taken by the oral specialist to review the images through the computer. There is a need to train dentists in taking high-quality order graphs. Some studies were similar to other studies [26, 59], where moderate to strong concordance was seen between clinical examination and evaluation of the images taken through mobile phones in identifying the presence of various types of lesion and the decision to refer or not. It was found that this workflow assisted in screening patients at the community level and their follow-up [42]. Most of the examiners used powder-free gloves so that they did not interfere with the long-term usage of the phone [42]. This workflow of screening High-risk lesions with the help of mobile phones is emerging as a great oral cancer surveillance tool. There is a need to include detailed information on patient history, good quality measures, and adequate training for the use of mobile phone cameras. 8 or 13-megapixel cameras gave the best images compared to five-megapixel cameras [42].

### 4.3. Barriers to Oral Cancer Screening Using mHealth

Reported barriers to screening included social factors, cultural limitations, and financial constraints [49]. This included significant problem challenges for patients such as difficulty in getting permission to leave work, the expense of traveling, and loss of daily wage when the participants take leave to attend the hospital appointment [49]. Several implementation challenges [48] included the involvement and interest of local authorities, the need for a standard training module, unexpected delays when frontline health professionals dropped out after training, inability to attend when male members of the family were not present, social stigmas, insufficient time due to work commitments, misconception such as “tobacco gives relief from toothache,” self-management of oral lesions by taking “more lime” to regress, and poor lighting within homes for proper screening. Other reasons included poor compliance to recall due to unwillingness to travel the distance for a biopsy, unwillingness to biopsy due to fear of injections, blood, and formation of the wound. There were some technical challenges too that affected the success of this mHealth initiative, such as the need for a minimum size of the photos, capturing only the positive sites, variation in the quality of images based on the distance of the camera, and zooming of the screen, and training of examiners before taking photographs.

### 4.4. Technical Challenges in the Implementation of mHealth

Since mHealth is solely based on the application of technical knowledge for health care, various technical challenges have been reported [60]. For example, the font size of the text seen on the mobile phone was not satisfactory for some participants as they believed the paper-based system had better visibility. Similarly, the screen size, display setting, and the ability of the participant to use mobile cell phones for reasons other than attending phone calls were also significant challenges. Acceptance of mHealth technology was also dependent on the level of education [61]. Moreover, there is a need to correctly train the participants regarding data security and privacy, using the technology, and managing data. Participants with insufficient training could find it cumbersome to use the mHealth technology. The lack of secured wireless networks for many physicians to send sensitive patient information can result in a breach of data privacy and endanger the security of one's medical data. Poor internet connectivity in resource-restricted areas is also one of the major technical challenges that need attention.

### 4.5. Limitations of the Included Studies

Some studies did not calculate the intra-observer agreement between the community health workers in detecting normal and abnormal oral lesions and power calculation [35, 42, 48]. Most of the participants all belonged to the same geographic region, so this study's findings cannot be generalized. Difficulty in retraction during the oral examination can interfere with the findings [35, 38]. The mobile phone applications collected only a limited number of clinical information, such as risk habits, clinical signs, and patient symptoms. However, information about past illness, comorbidities, intake of medication, and previous treatment history was not considered, which could have affected the rate of referral decision [35, 38, 40]. Moreover, there was no standardization of images in terms of lighting and angle of the image taken at different visits. Another limitation was the use of natural light to examine oral lesions [41]. The movement of the subject or phone resulted in some of the images having poor quality [38]. Compressing images while sending through WhatsApp might also have affected the diagnosis [40]. Some study literate patients full new to using the mobile application were selected [28].

Furthermore, a lack of technological expertise in the dentist may also make mHealth slow [41]. Certain issues related to handling and exporting files in the app were also reported [41]. Other issues faced in this study include lack of space storage in the mobile phone due to many videos [41]. Intrinsic limitations mentioned in this study include insufficient time to train the dentist to use mobile phone cameras [42]. This specialist might remember the diagnosis made during an oral clinical examination [42].

### 4.6. Future Perspectives

The sample size and intraexaminer agreement should be calculated for every study. Various geographical areas should be included in the studies in order to generalize the results. Better infrastructure including sufficient sterilized instruments, uninterrupted internet connection, expertise for optimization of the captured image, facility to upload image in low bandwidth of Internet connectivity, use of a magnifying lens and better lighting, affordable smartphones, provision to include detailed past, present, and medical history of the patients. The applicability of this mobile app among illiterate people and people from low socioeconomic status needs to be further studied. Technological expertise on the part of the dentist will help. In countries like Australia, telemedicine is being used more and more to provide early detection and family follow-up in cancer [62]. There is a lack of studies that report mHealth in oral cancer screening from the middle east region. Hence, studies to explore the feasibility of this technique in the Arab region should be planned. mHealth can also be used as a screening option in case of a pandemic scenario where face-to-face clinical examination might not be feasible [63]. Not only for diagnosis, mHealth can also be used to reduce patient care needs and improve the quality of life after oral cancer surgery [64]. mHealth and telemedicine are interesting and promising technologies. Nonetheless, it is still unclear whether rural settings in resource-restricted regions have sufficient infrastructure and uninterrupted Internet connectivity to adapt this diagnostic pathway.

## 5. Conclusion

Existing evidence shows mHealth to be useful for oral cancer early detection with good diagnostic accuracies. Its effectiveness as a potential tool for the early detection of oral cancer is increasing due to the access to mobile phones in all socioeconomic strata. Still more needs to be done in standardization and establishing a workflow to use it in routine clinical practice and community health centers for remote diagnosis/consultation. There are some barriers such as social factors, cultural limitations, technological, and financial constraints. Although existing literature points toward the feasibility of the early detection of oral cancer, there needs to be an improvement in the effectiveness of existing mHealth approaches and the development of new mHealth-based systems.

## Figures and Tables

**Table 1:** Clinical studies reporting mHealth in oral cancer screening.

Sl no	Author and region	Number of participants screened	Level of agreement and diagnostic accuracy among examiners of mHealth program	If mHealth is a feasible option for early oral cancer screening
		mHealth application used		
1	Ramesh et al. [34] India	2,686	Moderate to high diagnostic accuracy (ranging from 70.3% to 89.9%)	Yes, in resource-restricted and remote settings
		Not available		

2	Thampi et al. [35] India	1200	Very high agreement (*κ* = 0.9) overall sensitivity of 96.69% and specificity of identification of 98.69%	Yes, in resource-restricted and remote settings
		Name of the application used not available		

3	Haron et al. [36] Malaysia	355	Moderate to high agreement (*κ* = 0.604 and 0.892, respectively). High sensitivity (94.0%), specificity (95.5%) and inter-rater agreement for a referral decision (0.825)	Yes, in resource-restricted and remote settings
		MeMoSA		

4	Song et al. [37] India	5,025	Overall high accuracy (81%) in discriminating between normal/benign lesions	Yes, in resource-restricted and remote settings
		Deep learning-based MobileNet integrated into a mobile application		

5	Birur et al. [38] India	50	Moderate (*κ* = 0.62) to near-perfect agreement (*κ* = 0.92). Diagnostic accuracy ranged from 84.7% to 97.7%	Yes, in resource-restricted and remote settings
		Poi mapper		

6	Deshpande et al. [28] India	50	Overall, very positive acceptance	Yes, in resource-restricted and remote settings
		Prayaas		

7	Sunny et al. [39] India	82	Moderate level of agreement (*κ* = 0.67 to 0.72). Overall accuracy of 84% to 86% low sensitivity (18%)	Yes
		Cellscope		

8	Vinayagamoorthy et al. [40] India	131	Moderate reliability (*κ* = 0.59 and 0.55, respectively, for 2 examiners)	Yes, in resource-restricted and remote settings
		WhatsApp		

9	Gomes et al. [41] Brazil	55	Moderate agreement (*κ* = 0.597). High sensitivity (average 91%), specificity (average 90.5%), and accuracy (average 90.90%)	Yes
		Yes, but name is not available		

10	Haron et al. [42] Malaysia	16	Moderate to strong agreement (*κ* = 0.64 to 1.00). Overall good sensitivity (70%) and high specificity (100%)	Yes
		Mobile phone images		

11	Birur et al. [33] India	3,440	In the targeted cohort, there were 61% interpretable images, and 45% of the lesions were confirmed by specialists. The opportunistic cohort showed 100% concordance with the specialists	Yes, in resource-restricted and remote settings
		Oncogrid		

12	Praveen et al. [43] India	1,357	Use of mHealth enabled in an electronic record of subject details that can be used for a planned follow-up of the same cohort	Yes, in resource-restricted and remote settings
		Poi mapper		

**Table 2:** Studies mentioning development of device developments.

Sl no	Author and region	Outcome related to early oral cancer screening using mHealth program
1	Uthoff et al. [45] India	Cloud-based remote specialist and convolutional neural network clinical diagnosis allow for both remote community and home use when the flexible, dual-modality smartphone-based handheld probe is used
2	Uthoff et al. [31] India	This system showed high accuracies (81.25% to 94.94%) in classifying lesions into “suspicious” and “not suspicious”
3	Skandarajah et al. [46] India	Mobile microscopy showed agreement with existing oral cancer screening techniques
4	Yu et al. [47] US	This innovative tool eliminated operator bias, reduced size and power consumption when used for the diagnosis of early premalignant lesions

**Table 3:** Qualitative studies reporting the suitability of mHealth for early screening of oral cancer.

Sl no	Author and region	App used for training	Outcome related to early oral cancer screening using mHealth program
1	Subramaniun et al. [52] India	M-OncoED	Demonstrated the applicability of using an mHealth app to educate physicians
2	Haron et al. [50] Malaysia	MeMoSA	The app aided in identifying oral mucosal lesions
3	Bhatt et al. [49] India	Medic mobile	The system was accepted by frontline health care providers and enhanced their social standing
4	Chigurupati et al. [48] India	Poi mapper, sana	mHealth-based approach can aid remote early detection of oral cancer in resource-constrained settings
5	Hariprasad et al. [51] India	ECHO	A significant increase in the score of knowledge was found after the initial in-person training using the ECHO platform

## Abbreviations

(mHealth): Mobile health
(MeSH): Medical subject headings
(app): Mobile application
(*κ* value): Kappa value of agreement
(*χ*^2^-value): Chi-Square value
(95% CI): 95% Confidence interval
*(MeMoSA)*: Mobile mouth screening anywhere
(ANN): Artificial neural network
(CNN): Convolutional neural network
(FHW): Frontline health care workers
(LED): Light-Emitting diode
(AFI): Autofluorescence imaging
(WLI): White light imaging
(ROC curve): Receiver operating characteristic curve
(DRS): Diffuse reflectance spectroscopy
(ECHO): Extension for community health outcome
(KB): Kilobytes.
